# Loss of the plastidial signaling molecule, ppGpp, accumulation alters nuclear gene expression during nitrogen starvation

**DOI:** 10.3389/fpls.2026.1775338

**Published:** 2026-04-20

**Authors:** Takanari Nemoto, Yuto Omata, Masataka Inazu, Kazuma Sakoda, Atsushi Sakurai, Sousuke Imamura, Shouta Nonoyama, Shinji Masuda

**Affiliations:** 1 Department of Life Science and Technology, Tokyo Institute of Technology, Yokohama, Japan; 2 Department of Life Science and Technology, Institute of Science Tokyo, Yokohama, Japan; 3 Space Environment and Energy Laboratories, NTT, Inc., Musashino-shi, Tokyo, Japan

**Keywords:** Arabidopsis, chloroplast, nitrogen starvation, ppGpp, retrograde signal

## Abstract

Nitrogen (N) deficiency triggers major transcriptional reprogramming in plants. The chloroplast alarmone, guanosine tetraphosphate (ppGpp) synthesized and hydrolyzed by RelA-SpoT homologs (RSHs), has been proposed to regulate cellular metabolism. Here, we characterized an Arabidopsis mutant lacking all *RSHs* (*quadruple*), which accumulates no detectable ppGpp. Transcriptome analysis showed that 774 and 2,928 nuclear-encoded genes were differentially expressed in *quadruple* compared with the wild type (WT), under +N and -N conditions, respectively. Upon transition from +N to -N conditions, 2,487 nuclear genes in WT and 1,505 in *quadruple* primarily associated with cell wall biosynthesis and defense responses, were differentially expressed, suggesting that plastidial ppGpp is involved in the reprogramming of nuclear gene expression in response to N availability. Network analysis of transcription factors (TFs) indicated that ppGpp alters TFs expression and identified 11 candidates of master regulator of TFs expression by ppGpp-dependent manner during N starvation. Transcript levels of several plastid-encoded genes were higher in *quadruple* compared to WT under -N conditions, whereas mitochondrial transcripts were less affected. Together, these findings suggest that ppGpp acts as both a regulator and a key component of retrograde signaling, coordinating nuclear transcription and metabolic adaptation in response to N availability.

## Introduction

Nitrogen (N) is a fundamental macronutrient for plant growth and its availability often limits productivity in ecosystems and agriculture (Kant et al., 2011). Plants adapt to fluctuating N availability by reprogramming metabolism and gene expression, downregulating growth processes and activating survival pathways such as autophagy and internal N remobilization (Malagoli et al., 2005; Masclaux-Daubresse et al., 2010; Krapp et al., 2011). One of the central regulators of N-stress adaptation is guanosine tetraphosphate (ppGpp), a well-known second messenger in bacteria (Potrykus and Cashel, 2008; Corrigan et al., 2013) and plastids (Avilan et al., 2021; Boniecka et al., 2017; Braeken et al., 2006; Field, 2018; Imamura et al., 2018; Liu et al., 2025; Masuda et al., 2008; Mehrez et al., 2023; Mu et al., 2023; Potrykus and Cashel, 2008; Sugliani et al., 2016; Witte and Herde, 2024). In *E. coli*, ppGpp is synthesized by RelA and SpoT and modulates metabolism in response to environmental stress such as nutrient starvation (Potrykus and Cashel, 2008; Corrigan et al., 2013). In plants and algae, ppGpp is synthesized and hydrolyzed by nuclear-encoded RelA-SpoT homologs (RSHs), which are localized in chloroplasts (Masuda et al., 2008; Sugliani et al., 2016; Boniecka et al., 2017; Imamura et al., 2018; Avilan et al., 2021; Mehrez et al., 2023). *Arabidopsis thaliana* has four RSHs: RSH1, RSH2, RSH3, and Ca^2+^-activated RSH (CRSH) (Ono et al., 2021). Previous studies have shown that plastidial ppGpp represses chloroplast-encoded gene expression, leading to reduced photosynthetic activity and enhanced stress tolerance under diverse environmental conditions (Maekawa et al., 2015; Field, 2018; Li et al., 2022; Romand et al., 2022; Wang et al., 2023), indicating the important role of ppGpp during plant responses to N starvation.

Arabidopsis mutants lacking RSH2 and RSH3 fail to downregulate chloroplast-encoded genes under –N conditions, although exact ppGpp levels under –N conditions were not examined (Honoki et al., 2018; Goto et al., 2022). An Arabidopsis mutant lacking all four RSHs (*quadruple*), accumulating no detectable ppGpp, exhibits altered expression of both plastid- and some nuclear-encoded genes (Inazu et al., 2024). Similarly, Arabidopsis mutants with reduced ppGpp accumulation (~10% of WT) exhibit broad nuclear transcriptional changes and impaired responses to nitrogen deficiency (Romand et al., 2022). Relationship between ppGpp-dependent regulation of plastidial metabolism and cytosolic nutrient responses mediated by the target of rapamycin (TOR) kinase has been also reported (Imamura et al., 2018; Crespo et al., 2025). These findings suggest that ppGpp homeostasis in plastids has an important role for stress acclimation of plants, and ppGpp may function not only as a stress signal but also as a basal regulator of plant metabolism. However, impact of the complete loss of ppGpp synthesis in plastids on nuclear gene expression has not yet been elucidated.

Here, we conducted transcriptome analysis of *quadruple* of Arabidopsis to evaluate the effect of complete loss of RSH-dependent ppGpp synthesis under –N conditions. The results showed that plastidial ppGpp influences on global transcription reprograming of both nuclear and plastid genomes during –N response. We also found transcription factors mediating the response. A model of the potential reprogramming events that occur after the early vegetative phase, which might better reflect the physiological processes underlying long-term adaptation to N starvation is proposed.

## Materials and methods

### Plant growth conditions

*Arabidopsis thaliana* (*Columbia* ecotype) and its corresponding mutants were subjected to surface sterilization followed by vernalization. This process entailed a 4-day exposure to 4 °C in complete darkness. After vernalization, seeds were plated on 1×Murashige and Skoog (MS) medium containing 0.8% INA-AGAR (Funakoshi, Tokyo, Japan) and 1% (w/v) sucrose at 23°C under continuous light conditions (40 µmol photons m^-2^ s^-1^). For N-deficient (–N) medium, KNO_3_ was replaced with KCl with the same concentration, all NH_4_NO_3_ was removed, and KNO_3_ and NH_4_NO_3_ were added to 0.1 mM each. The *rsh1-rsh2-rsh3-crsh* mutant, *quadruple*, was previously described (Inazu et al., 2024).

### Anthocyanin content measurement

Anthocyanin content was determined following the method of Lange et al. (1971). The shoots of individual seedlings were weighed (Fresh Weight, FW) and homogenized in 250 μL of extraction solvent (propanol:HCl:H₂O = 18:1:81, v/v/v). The samples were boiled for 3 min and centrifuged at 15,000 rpm for 5 min. Absorbance of the supernatant was recorded at 535 nm and 650 nm using a UV-1800 spectrophotometer (Shimadzu, Japan). To compensate for scattering, the relative anthocyanin level was calculated as (*A*535−2.2×*A*650).

#### RNA purification

Total RNA was extracted from shoots of plants grown on MS medium using the SV Total RNA Isolation System (Promega, Madison, WI, USA), according to the manufacturer’s instructions. For each genotype and treatment condition, RNA was isolated from four independent biological replicates. Equal amounts of RNA from these replicates were pooled to generate a single representative sample per group. RNA quality was assessed using Agilent 2100 Bioanalyzer system (Agilent Technologies, Santa Clara, CA, USA), which were outsourced to GeneBay (Yokohama, Japan), and samples with RNA integrity number (RIN) values ≥ 6.5, according to the analysis company’s criteria, were used for subsequent RNA-seq analysis. Following ribosomal RNA depletion, RNA sequencing libraries were constructed and sequenced, which were outsourced to GeneBay.

#### RNA sequencing, and data processing

The raw paired-end reads were aligned to the *A. thaliana* reference genome using STAR (v2.7.9a) [2]. Gene- and transcript-level expression quantification and normalization were performed using RSEM (v1.3.3) [3]. Differentially expressed genes (DEGs) were identified using thresholds of |log_2_ fold change| > 2 and *p*-value < 0.01. GO analysis was conducted using R package “clusterProfiler” (Yu et al., 2012). KEGG pathways for each gene were annotated using R package “KEGGREST”. Biological process was selected for GO terms (**Supplementary Table 1**). For transcription factors’ network, TF2 Network (http://bioinformatics.psb.ugent.be/webtools/TF2Network/) was used (Kulkarni et al., 2018). Transcription factors were selected according to the list available in TF2Network. Transcription factors in DEGs comparison under nitrogen sufficient condition and deficient condition in WT were extracted and TF2Network analysis have conducted (**Supplementary Table 1**). The exported network was then visualized with Cytoscape (Shannon et al., 2003). RNA-seq raw data are available at the DDBJ Sequence Read Archive (BioProject ID: PRJDB37604).

### Quantitative real-time PCR

Plants were grown under nitrogen sufficient condition for 14 days and transferred to +N and/or –N conditions and grown for 10 days. Then the plant shoot samples were collected and frozen with liquid nitrogen. The samples were then homogenized, and the total RNA were extracted with the SV Total RNA Isolation System (Promega). Total RNA was then reverse transcribed with PrimeScript RT reagent kit (TaKaRa, Kyoto, Japan) with manufactures instruction. Primers for analyzing transcript levels of *UBQ10* (5’-GGCCTTGTATAATCCCTGATGAATAAG-3’ and 5’-AAAGAGATAACAGGAACGGAAACATA-3’), *PDF1.1* (5’-CCTTCCTTTTCGCTGCTCTTG-3’ and 5’-TTACTGTTTCCGCAAACGCCTG-3’), COG1 (5’-TGCGACTGGAGCTGTTGATC-3’ and 5’-CCGTAGCAGCTCTCCACTCTTC-3’) and *LHCB1.1* (5’-TCACGCTCAGAGCATTTTGG-3’ and 5’-CCCATTTCCTGCGACTCTGT-3’) are used.

### Statistical analysis

Statistical analyses of qRT-PCR data were performed using Welch’s *t*-test, which accounts for unequal variances between groups. A two-tailed *p*-value < 0.05 was considered statistically significant. Data are presented as mean ± standard deviation (SD) of biological replicates.

## Results

### Nuclear gene expression is influenced by the level of plastidial ppGpp

**Figure 1:**
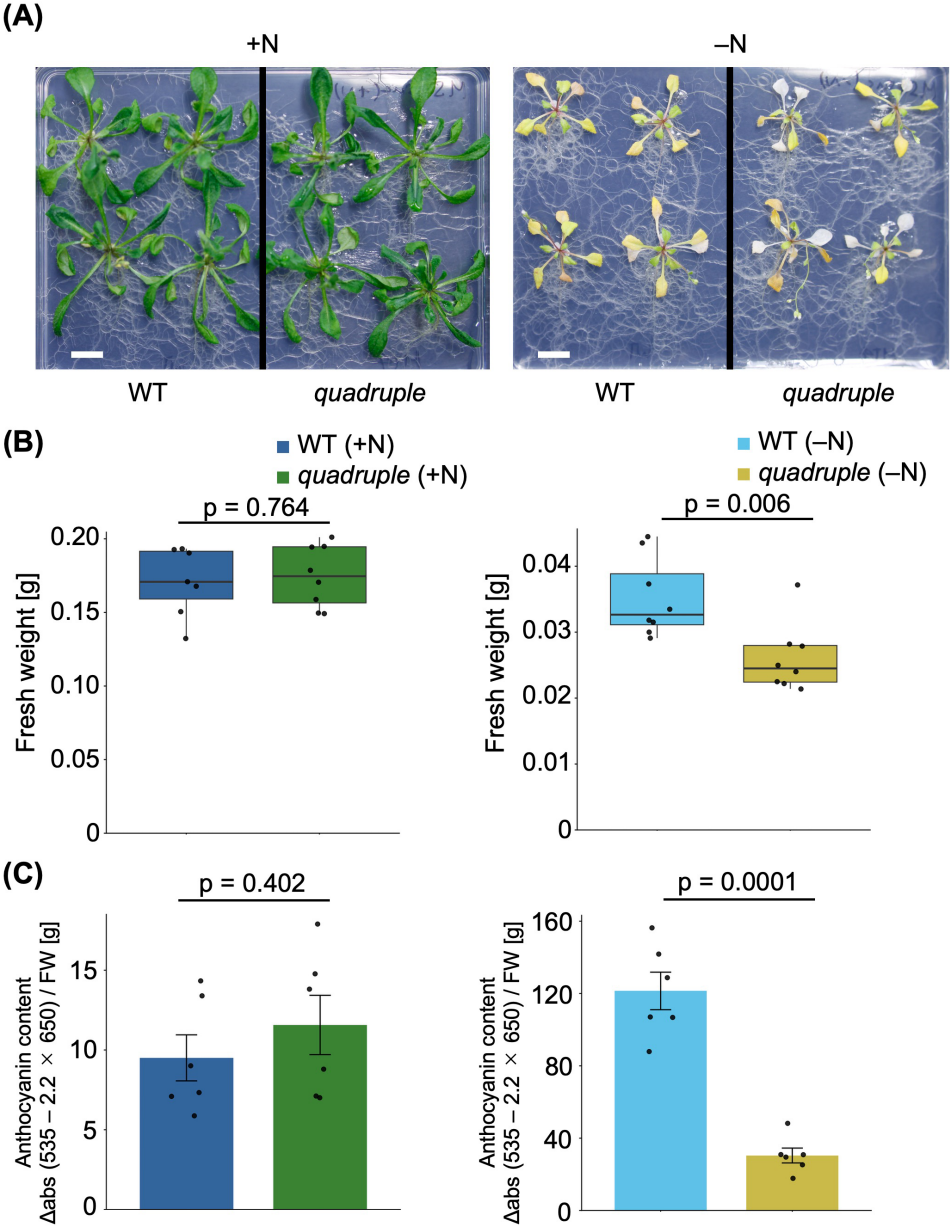
**(A)** Phenotype of plants grown under N-sufficient conditions for 14 days followed by 10 days under N-sufficient or N-deficient conditions. **(B)** Fresh weight of the plants shown in panel **(A)**. **(C)** Anthocyanin content of plants grown under the same conditions as in panel **(A)**. Error bars represent SE (n = 3). P-values are shown above each graph (Welch’s t-test).

To investigate how the plastidial ppGpp contributes to transcriptional regulation in the nucleus, plastids and mitochondria, we utilized the Arabidopsis *quadruple* mutant, in which all ppGpp synthases and hydrolases are eliminated, resulting in no detectable ppGpp accumulation (Inazu et al., 2024). This mutant enables us to investigate the physiological role of ppGpp in the transcriptional regulatory network in plant cells. WT and *quadruple* plants were grown on MS plates for 14 days, then transferred to –N or control MS (+N) plates for an additional 10 days. A previous study showed that in WT, ppGpp increased ~5-fold after 4 days of N starvation and remained ~2-fold elevated for at least 12 days, while no ppGpp was detected in the *quadruple* (Inazu et al., 2024). Under these conditions, no obvious differences in overall growth were observed between WT and *quadruple* on +N plates. In contrast, under –N conditions, *quadruple* plants exhibited earlier senescence than WT (**Figure 1A**). Consistent with this phenotype, fresh weight was significantly reduced in *quadruple* under –N conditions (**Figure 1B**). Notably, anthocyanin accumulation, which is typically induced under stress, was significantly lower in *quadruple* than in WT under –N conditions (**Figure 1C**). Total RNA was extracted from shoots harvested from plants grown under these conditions and subjected to RNA sequencing.

We first verified the reliability of the RNA sequencing data by conducting qRT-PCR analysis. The RNA-sequencing dataset indicated that transcript levels of *plant defensin 1.2* (*PDF1.2*) were markedly reduced in both WT and *quadruple* upon transition from +N to –N conditions (log_2_ fold change, log_2_FC = –6.95 and –1.69, respectively). Consistently, the qRT-PCR analysis showed that transcript levels of *PDF1.2* in WT and the *quadruple* decreased to 0.56% (± 1.15%, *n* = 3) and 0.22% (± 1.2%, *n* = 3) of those under +N conditions, respectively, thereby supporting the reliability of the RNA-sequencing data. To confirm that the plants were responding to nitrogen deficiency, we checked the transcript levels of known nitrogen starvation-responsive genes. The expression levels of several N-deficiency responsive genes, including *NRT2.5*, which is known to be induced under nitrogen-deficient conditions (Lezhneva et al., 2014), were increased in both WT (log2FC = 2.49) and *quadruple* (log2FC = 2.76).

To examine global transcriptomic differences between WT and *quadruple*, RNA-seq data was subjected to principal component analysis (PCA) (**Supplementary Figure 1**). The PCA analysis showed that the transcriptomic features were separated samples by nitrogen availability. Specifically, different growth conditions (+N and –N) were separated in component 1, whereas the genetic backgrounds of the WT and *quadruple* mutant were separated in component 2. Consequently, the separation between WT and *quadruple* mutant was relatively larger under –N conditions than under +N conditions. These results suggest that the function of four RSHs is required for transcriptional regulation not only under −N conditions but also under +N conditions, and it is particularly required for transcriptomic changes induced by the transition from +N to –N conditions.

**Figure 2:**
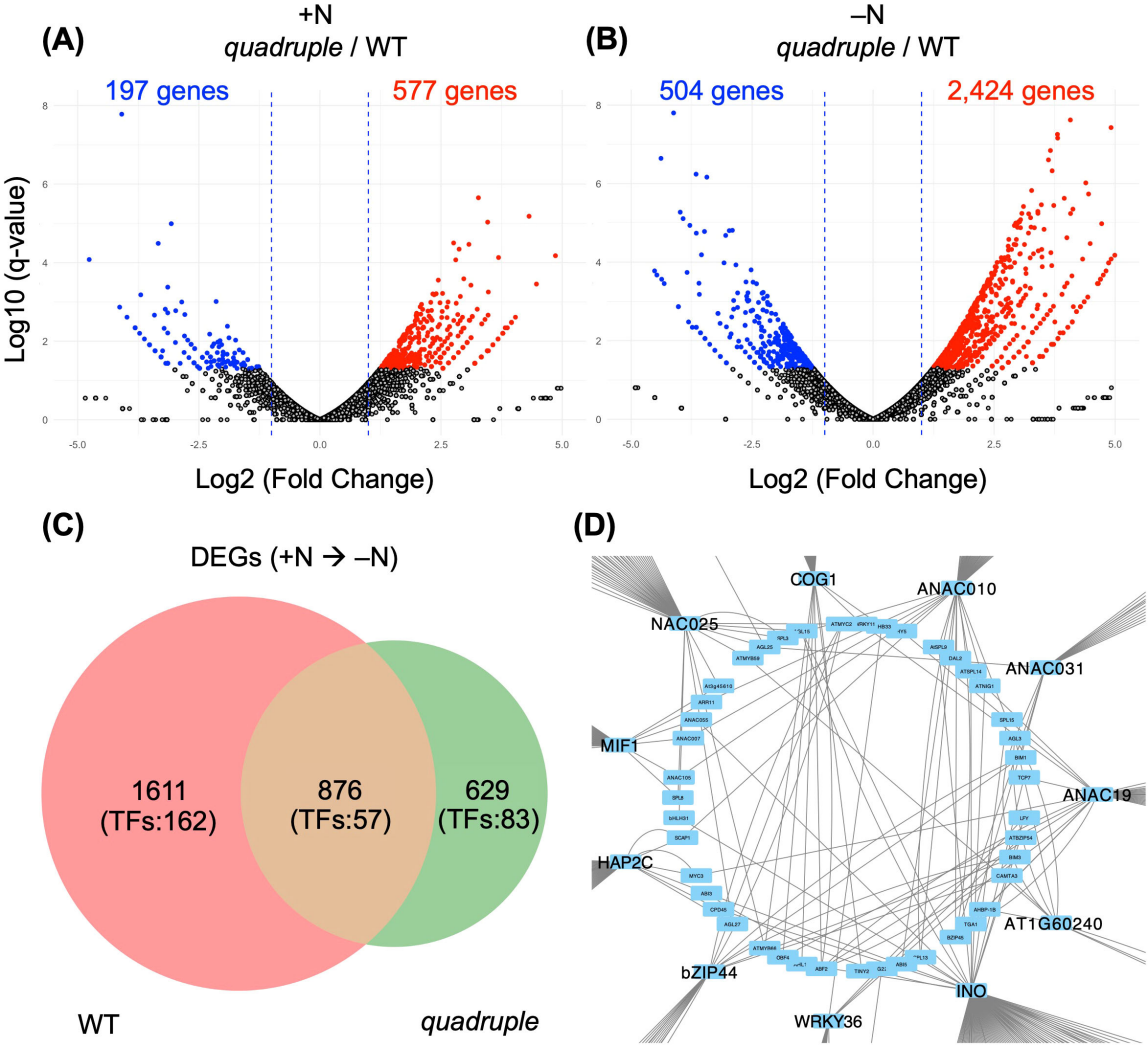
**(A, B)** Volcano plots comparing nuclear gene expression profiles between WT and *quadruple* under N-sufficient and N-deficient conditions. **(C)** Venn diagram of differentially expressed genes (DEGs) under nitrogen-deficient conditions in each genotype. The number of transcription factors (TFs) among unique DEGs is indicated. A total of 876 genes were shared between nitrogen-responsive genes in WT and the *quadruple* mutant, and this overlap was significantly greater than expected by chance (hypergeometric test, P < 1 × 10^-300^). **(D)** Network of TFs upregulated in WT under nitrogen-deficient conditions. The complete network is shown in **Supplementary Figure 2**.

Comparative analysis of the RNA-sequencing data of nuclear-encoded genes identified 774 DEGs between WT and *quadruple* under +N conditions (**Figure 2A**). Specifically, transcript levels of 197 and 577 genes were significantly reduced and increased, respectively in *quadruple* than WT. This suggests that ppGpp exerts a regulatory influence on basal gene expression even under +N conditions, although no visible phenotypic differences were observed between the two lines at the growth conditions (Inazu et al., 2024). It should be noted that because the plants were transplanted from +N to –N conditions, it is inevitable that they experienced mechanical stress; however, since plants transferred from +N to +N were used as the control, we believe that this effect is not reflected in the DEGs. Under –N conditions, more drastic differences were observed in the transcript levels between WT and *quadruple* than those under +N conditions: a total of 2,928 DEGs were detected between the two lines (**Figure 2B**). Specifically, transcript levels of 504 and 2,424 genes were significantly reduced and increased, respectively in *quadruple* than WT at the conditions. Many of the genes that were up- or down-regulated under +N and –N conditions also showed expression changes in response to nitrogen starvation alone, suggesting that most ppGpp-dependent transcriptional changes occur specifically under nitrogen-deficient conditions (**Supplementary Figure 2**). Interestingly, 176 genes were commonly upregulated and 19 genes were commonly downregulated irrespective of nitrogen availability, suggesting that these genes are regulated by basal ppGpp levels under steady-state conditions. The overlap between the DEG sets identified under +N and –N conditions was highly significant (**Supplementary Figure 2**; hypergeometric test: upregulated genes, P = 9.35 × 10^-64^; downregulated genes, P = 1.78 × 10^-10^). These results indicate the importance of RSH-mediated ppGpp synthesis/hydrolase in plastids for extensive transcriptional reprogramming in response to N starvation. Importantly, approximately 82% of the DEGs under –N conditions were upregulated in *quadruple* compared with WT, suggesting that ppGpp plays a more critical role in mediating transcriptional repression than in promoting activation of nuclear genes in response to N starvation. This suggests that plastidial ppGpp downregulates transcription in the nucleus for resource reallocation under adverse conditions. Given basal levels of ppGpp may serve to fine-tune chloroplast function and maintain metabolic homeostasis (Sugliani et al., 2016; Romand et al., 2022), the results suggest a dual role of ppGpp in both stress-induced transcriptional repression and basal metabolic regulation across vegetative-growth stages.

Since the alteration of transcript levels caused by the *quadruple* mutation was enhanced under –N conditions (**Figure 2B**), we hypothesize that ppGpp is required for the proper initiation and propagation of nuclear transcription responses induced by N starvation. In fact, although WT plants exhibited 2,387 DEGs between +N and –N conditions, only 1,505 DEGs were identified in *quadruple* (**Figure 2C**, **Supplementary Table 1**). Of these DEGs, 876 were shared between WT and the *quadruple*, and this overlap was highly significant according to a hypergeometric test (P < 1 × 10^-300^; **Figure 2C**). In contrast, nearly twice as many genes (1,611 genes) were uniquely differentially expressed in WT. These findings suggest that the loss of ppGpp impairs the ability to dynamically reprogram gene expression in response to N-starvation stress, resulting in an overall alteration in transcriptional activity.

**Figure 3:**
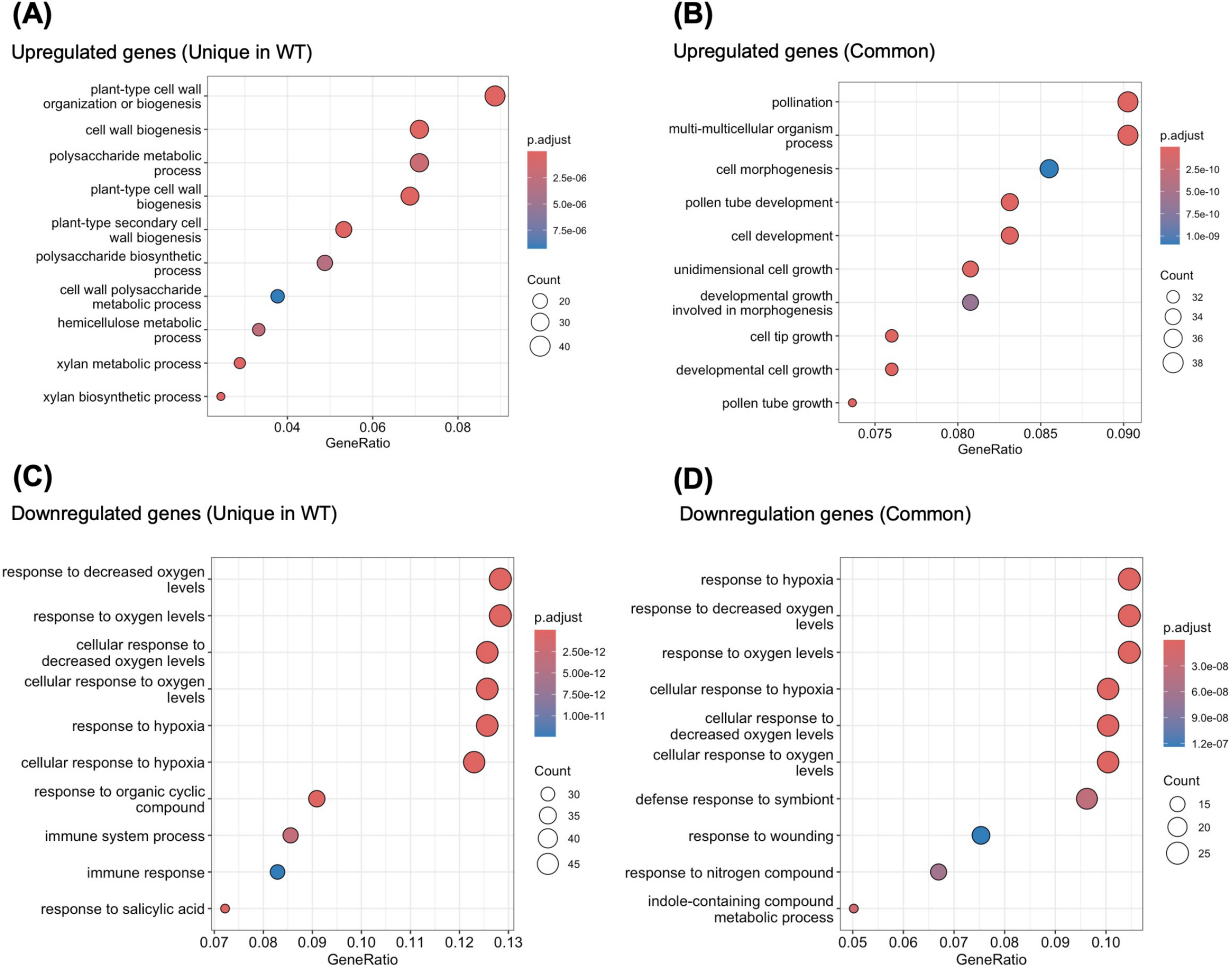
GO enrichment analysis for DEGs under N-deficient conditions. **(A)** DEGs upregulated in WT. **(B)** DEGs upregulated in both WT and *quadruple*. **(C)** DEGs downregulated in WT. **(D)** DEGs downregulated in both WT and *quadruple*.

To gain further functional insight into the ppGpp-dependent regulation of nuclear gene expression, we performed Gene Ontology (GO) enrichment analysis on genes that were upregulated in WT following a shift from +N to –N conditions, but were not differentially expressed in *quadruple* under the same conditions. Among the upregulated genes, we observed significant enrichment of GO terms related to cell wall dynamics, including plant-type cell wall biogenesis (e.g., *CESA4*), and *xylan metabolic process* (e.g., *GXM1*) (**Figure 3A**, **Supplementary Table 1**). These biological processes are typically associated with structural reorganization of cell walls, which plays a central role in environmental acclimation (Landi and Esposito, 2017; Qin et al., 2018; Tian et al., 2022; Lu et al., 2025). Under –N conditions, plants must reallocate carbon resources away from rapid growth toward adaptive remodeling, including reinforcement of cell walls and modulation of intercellular signaling (Tenhaken, 2014), suggesting that ppGpp is indispensable for activating these biosynthetic pathways, and its absence results in a failure to execute structural adaptation at the cellular level.

In contrast, GO terms enriched in the upregulated DEGs in both WT and *quadruple* were dominated by broader developmental categories such as pollen tube development (e.g., *EDA5*), cell morphogenesis (e.g., *RIC1*), and unidimensional cell growth (e.g., *GA20OX2*) (**Figure 3B**). These results suggest that some basal developmental programs remain active regardless of ppGpp. GO analysis of downregulated genes provides further support for the centrality of ppGpp in stress-induced transcriptional suppression. Among WT-specific downregulated DEGs, enriched GO terms included response to hypoxia (ex. *EXL1*), immune system process (*NUDT4*), and response to salicylic acid (*WRKY70*) (**Figure 3C**). These pathways are energetically costly and often repressed under nutrient limitation to conserve energy and resources (Baena-González et al., 2007). The specific repression of these pathways in WT, but not in *quadruple*, suggests that ppGpp is required for initiating a global suppression of energy-intensive or defense-related responses during N-starvation stresses. It is notable that many of the GO terms enriched in WT-specific DEGs align with known N-starvation responses, such as hypoxia signaling and hormonal modulation (Krapp et al., 2011; Kiba et al., 2012). For example, the repression of hypoxia-responsive genes under N starvation in WT likely reflects a broader metabolic downshift aimed at reducing oxygen consumption in energy-limited conditions—a response absent in *quadruple*.

In the commonly downregulated DEGs of both WT and *quadruple* (**Figure 3D**; **Supplementary Table 1**), GO terms such as response to hypoxia (e.g., *LBD38*) and response to wounding (e.g., *WRKY40*) were still present, suggesting that certain aspects of the N-starvation response are at least partially independent of ppGpp. However, the overall number and enrichment strength of these terms were lower than those uniquely repressed in WT, reinforcing the idea that ppGpp enhances both the depth and specificity of transcriptional suppression. These results suggest that ppGpp enables plants to execute a bifurcated transcriptional strategy under –N conditions: (1) repressing energetically expensive pathways such as defense and oxygen-intensive metabolism, and (2) activating structural remodeling programs that facilitate stress acclimation. The impairment of both arms of this response in *quadruple* illustrates the central integrative function of ppGpp in coordinating metabolic and transcriptional adaptation.

Since several GO terms related to cellular metabolism were significantly enriched, we further analyzed genes associated with representative metabolic pathways, including glycolysis and the tricarboxylic acid (TCA) cycle. Among the genes upregulated in WT plants under –N conditions, several genes found were related to electron transport processes (e.g., *AHA8*) (**Supplementary Table 2**). In contrast, some genes involved in glycolysis (e.g., *FBA5*) were downregulated under the same conditions (**Supplementary Table 2**), suggesting the involvement of plastidial ppGpp for the regulation of the respiratory metabolism and that this change likely reflects the hypoxic response described above.

To explore potential signals linking plastidial ppGpp to nuclear transcriptional reprogramming under −N conditions, we analyzed the expression of ROS- and redox-related genes within the DEG sets. Several transcripts associated with ROS/redox processes were differentially expressed under −N. We observed the shared induction of antioxidant/redox factors such as *CSD1* and *CSD2*, together with genotype-specific expression changes in ROS- and redox-related regulators, notably repression of the NADPH oxidase *RBOHD*, in *quadruple* (**Supplementary Table 1**). Collectively, these results suggest that ppGpp deficiency influences the expression of a subset of ROS/redox-associated genes under nitrogen starvation.

### Transcription factors whose expression is influenced by N starvation and ppGpp

RNA-seq revealed that 219 transcription factor (TF) genes were differentially expressed in WT and 140 in *quadruple* upon transition from +N to –N conditions, with 57 shared between the two lines (**Figure 2C**; **Supplementary Table 1**). WT-specific upregulated TFs included WUS, NST1/2, and SND2/3, linked to floral organ development and cell wall modification (Villanueva et al., 1999; Lohmann et al., 2001; Mitsuda et al., 2005; Nakano et al., 2015). Nitrogen starvation induces biosynthesis of salicylic acid and other defense-related hormones (Conesa et al., 2020; Ding et al., 2021; Bagautdinova et al., 2022; Jia et al., 2022), which may stimulate wall remodeling, while promoting the shift from vegetative to reproductive growth (Sanagi et al., 2021; Cho et al., 2025). TFs often regulate expression of other TFs (Sun and Dinneny, 2018), suggesting the existence of a master regulatory mechanism underlying the –N responses. In order to identify potential master regulators of TFs, we constructed a transcriptional network based on the expression patterns of the TFs that were exclusively upregulated in WT under –N conditions (**Figure 2D**; **Supplementary Figure 3**; **Supplementary Table 1**). A transcriptional network of WT-specific TFs identified 11 candidates: COG1, NAC025, MIF1, AtHAP2C, AtZIP44, AtWRKY36, INO(SND3), AT1G60240, ANAC010, ANAC019, and ANAC031 (**Figure 2D**). Most of them are associated with stress responses and development, indicating that ppGpp activates an adaptive regulatory program.

**Figure 4:**
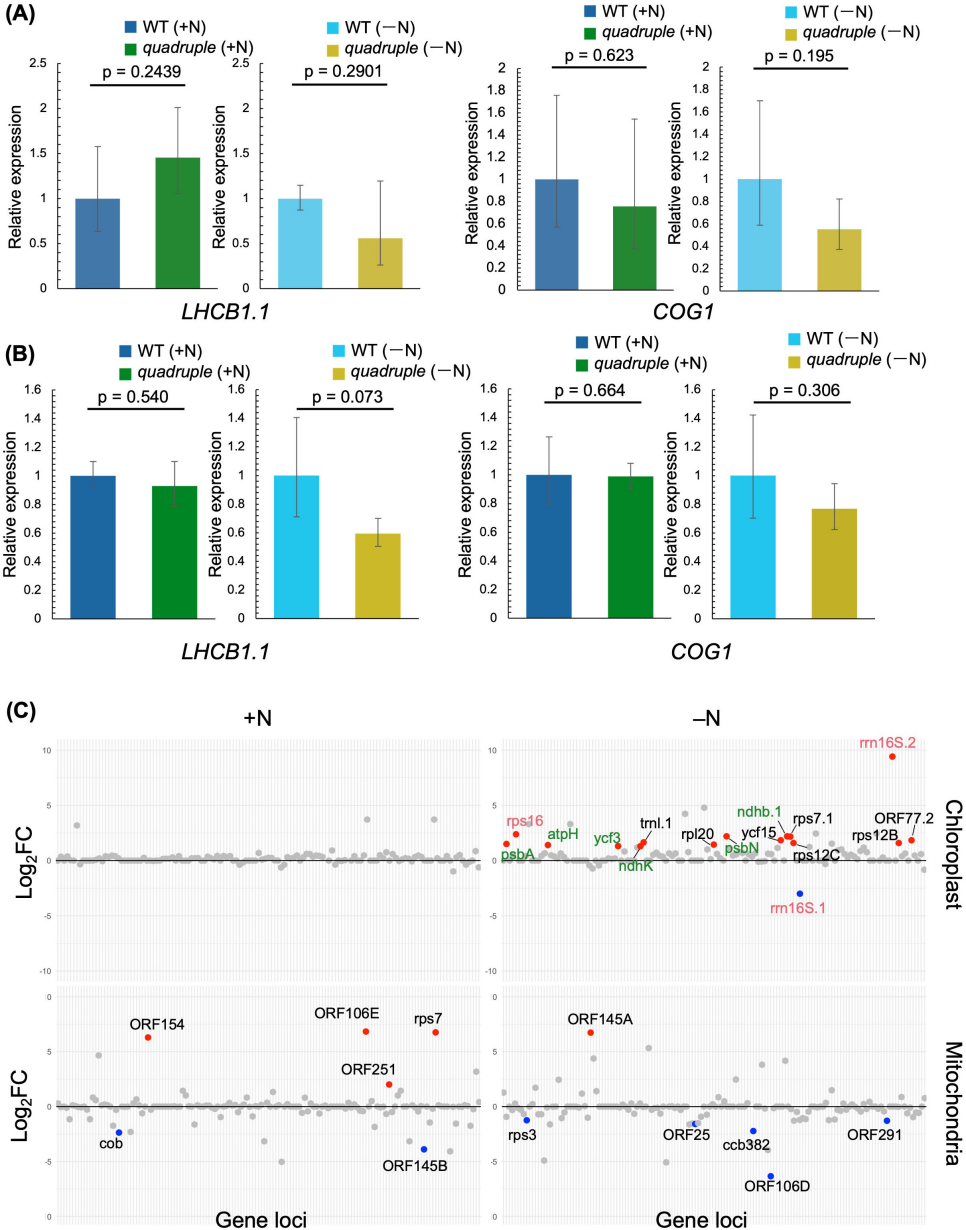
qRT-PCR analysis of *COG1* and *LHCB1.1*. **(A)** transcript levels in plants grown under +N conditions for 14 days, followed by 10 days of either +N or -N treatment. **(B)** transcript levels in plants grown under +N conditions for 14 days, followed by 14 days of either +N or -N treatment. **(C)** Manhattan plots of chloroplast and mitochondrial genes. The *x*-axis represents gene loci on the chloroplast or mitochondrial genomes, and the *y*-axis represents the log_2_ fold change in *quadruple* compared with WT for each gene. Red and blue dots indicate significantly upregulated and downregulated genes, respectively. For chloroplast genes, gene names highlighted in magenta correspond to NEP-transcribed genes, those in green correspond to PEP-transcribed genes, and black indicates genes with unknown transcriptional regulation. The log_2_ fold changes and associated p-values are provided in **Supplementary Table 1**.

Among the TFs, we focused on COG1, which regulates *LHCB1* and *LHCB2*, which encode light-harvesting chlorophyll-binding proteins essential for grana stacking and NPQ induction (Andersson et al., 2003; Wei et al., 2023), because the *quadruple* mutant was found to impair NPQ upregulation during the transition from +N to –N conditions (Inazu et al., 2024), suggesting that COG1-dependent transcriptional regulation seems to be involved in this processes. Specifically, we further examined *COG1* and *LHCB1.1* transcript levels by qRT-PCR (**Figures 4A, B**). For this analysis, plants were grown under +N conditions for 14 days, and then transferred to –N conditions followed by incubation either for 10 or 14 days. Both 10 and 14 days after transfer from +N to –N conditions, no significant differences were observed in the transcript levels of *COG1* and *LHCB1.1*; although both transcripts tended to be lower in *quadruple* compared with WT (*COG1* log_2_FC [*quadruple*/WT]: +N = –0.015, −N = –0.381; *LHCB1.1* log_2_FC [*quadruple*/WT]: +N = −0.105, −N = −0.751) (**Figures 4A, B**). These results suggest that prolonged nitrogen starvation promotes *COG1*-mediated upregulation of *LHCB1.1* in WT, thereby enhancing NPQ induction.

### Gene expression in chloroplasts and mitochondria under N-deficient conditions

We next investigated the organelle-specific transcript levels under –N conditions. Under +N conditions, no significant difference was observed in the transcript levels of chloroplast-encoded genes between WT and *quadruple* (**Figure 4C**; **Supplementary Table 1**). On the other hand, under –N conditions, transcript levels of 15 and 1 chloroplast-encoded genes were higher and lower in *quadruple* than WT, respectively (**Figure 4C**; **Supplementary Table 1**). Given that ppGpp accumulation downregulates chloroplast gene expression during N starvation (Li et al., 2022; Romand et al., 2022; Mehrez et al., 2023), these results suggest that WT represses chloroplast-encoded transcription under –N conditions, while this repression was largely absent in *quadruple*. Previous studies have demonstrated that ppGpp directly and/or indirectly inhibits activities of the plastid-encoded RNA polymerase (PEP) and the nucleus-encoded plastid RNA polymerase (NEP), leading to global suppression of photosynthetic and housekeeping genes encoded in the chloroplast genome (Maekawa et al., 2015; Romand et al., 2022; Inazu et al., 2024). This plastid-specific regulatory role of ppGpp has been corroborated by studies in *Arabidopsis* and *Marchantia*, where elevated ppGpp levels induce transcription reduction of plastid genes including those encoding photosystems, photosystem assembly factors and photosynthetic electron transfer components (Harchouni et al., 2022; Romand et al., 2022). The results of this study reinforce the model that N starvation induces ppGpp accumulation in plastids (Inazu et al., 2024), which in turn downregulates plastid gene expression.

We also observed that transcript levels of several mitochondrion-encoded genes differed between WT and *quadruple* under both +N and –N conditions (lower panels, **Figure 4C**), suggesting that plastidial ppGpp influences transcriptional control in mitochondria, although this regulation, if any, is not related to the –N response.

## Discussion

This study highlights the pivotal role of the chloroplast signaling molecule ppGpp in genome-wide transcriptional reprogramming of *Arabidopsis* during –N response. The results obtained were largely consistent with the previously reported RNA-seq data of ppGpp-accumulation-suppressed lines (Romand et al., 2022). Therefore, in the following sections, we focus our discussion on the findings revealed by the present results using the mutant completely lacking the ppGpp-synthesizing enzymes.

WT showed ~1.5-fold more DEGs than *quadruple* upon transition from +N to –N conditions (**Figure 2C**). GO enrichment of WT-specific DEGs highlighted cell wall remodeling, hormone signaling (ABA, SA), and reproductive development, consistent with known N-deficiency responses (**Figures 3A, C**) (Baena-González et al., 2007; Krapp et al., 2011; Kazan and Manners, 2013; Romand et al., 2022; Inazu et al., 2024). These systemic changes support a model in which ppGpp triggers retrograde signaling to coordinate nuclear transcription. Chloroplasts act as sensors and broadcasters of metabolic status, consistent with plastid-to-nucleus retrograde signaling (Woodson and Chory, 2008; Chi et al., 2015; Jan et al., 2022). Potential mediators include ROS, redox signals, and metabolites such as 3’-phosphoadenosine 5’-phosphate (PAP) (Estavillo et al., 2011; Chi et al., 2015; Dogra and Kim, 2020). Our results suggest that ppGpp accumulation under –N conditions perturbs chloroplast redox or metabolite balance, influencing the retrograde signaling to the nucleus. Notably, *quadruple* showed altered expression of ROS/redox regulators, including reduced transcript levels of *RBOHD*, together with changes in antioxidant/redox-associated genes (**Supplementary Table 1**), suggesting that ppGpp-induced changes in chloroplast metabolic state may intersect with redox/ROS and metabolite-mediated retrograde signaling pathways. Although we did not detect clear transcriptional signatures of canonical GUN1-dependent signaling in our dataset, the involvement of GUN1-related pathways cannot be excluded and may occur through post-transcriptional regulation or transient signaling events. Thus, ppGpp-dependent metabolic changes may converge with pathways such as SAL1-PAP, and potentially GUN1, to shape nuclear transcriptional responses during nitrogen starvation.

Importantly, however, this interpretation does not imply that the observed responses are simply a passive consequence of reduced energy demand under N starvation. Although nitrogen starvation generally reduces protein abundance and growth-related metabolic demand, this reduction is likely not merely a passive consequence of nutrient limitation. Because ppGpp represses chloroplast gene expression and photosynthetic functions, it may also contribute to the initial decline in chloroplast protein synthesis capacity, thereby promoting coordinated metabolic acclimation rather than simply reflecting reduced energy demand. Such coordinated acclimation is likely integrated with broader nutrient- and energy-signaling networks. A plausible hypothesis is that ppGpp intersects with nutrient-sensing kinases TOR and SnRK1. TOR negatively regulates ppGpp via RSH3 phosphorylation under +N conditions (D’Alessandro et al., 2024), while SnRK1 promotes autophagy during N starvation (Chen et al., 2017; Soto-Burgos and Bassham, 2017). Their convergence with ppGpp likely forms a multilayered system balancing growth and stress responses (Nukarinen et al., 2016; Dobrenel et al., 2025). Thus, ppGpp represses chloroplast transcription while activating retrograde signaling that reprograms nuclear gene expression through hormones and TF networks. WT-specific induction of TFs under –N conditions likely serves as central regulatory hubs; their absence in *quadruple* leads to impaired reprogramming and stress tolerance.

The expression levels of some chloroplast genes were repressed in WT, but not in *quadruple*, upon transition from +N to –N conditions, whereas mitochondrial expression was less affected (**Figure 4C**). Nevertheless, *quadruple* exhibited altered mitochondrial transcript levels under both +N and –N conditions (**Figure 4C**), and artificial ppGpp accumulation in mitochondria has been shown to induce growth retardation (Goto et al., 2024), suggesting that plastidial ppGpp may be involved in crosstalk between two organelles in an N-independent manner. Notably, *psbA* expression showed a significant decrease in WT in our RNA-seq dataset, although this difference was not detected in Inazu et al. (2024). This discrepancy likely reflects the larger biological variation observed in *quadruple* that reduced statistical power in qPCR-based analyses, as well as methodological differences in normalization procedures between RNA-seq and qPCR.

Network analysis suggests the involvement of the transcription factor COG1 for mediating chloroplast signals to nucleus to control photoprotective pathways (**Figure 2D**). WT plants showed more rapid NPQ induction than *quadruple* under –N conditions (van der Biezen, 2000; Masuda et al., 2008), although the mechanism behind the phenotype remains elusive. Given that COG1 upregulates the expression of *LHCB1* and *LHCB2*, which are important for NPQ induction (Andersson et al., 2003; Wei et al., 2023), our results suggest that NPQ induction under –N conditions is mediated by ppGpp-dependent activation of COG1 expression. In our experiment, although COG1 expression was strongly induced, *LHCB1.1* transcript levels did not show a clear change at the time of sampling. This may indicate that COG1 activation represents an early upstream regulatory event and that *LHCB1.1* upregulation occurs with a temporal delay. Previously, the GUN1-dependent retrograde-signaling also regulates *LHCB* expression under plastid dysfunction (Shai Koussevitzky et al., 2007; Chan et al., 2016). Perhaps, ppGpp accumulation perturbs plastid homeostasis, activating GUN1-dependent repression of *LHCBs*. Thus, *LHCB* expression might be under dual regulation: activation via COG1 and repression via GUN1. This layered mechanism likely fine-tunes light harvesting and NPQ induction during N deficiency, balancing photoprotection with photosynthetic efficiency.

In conclusion, plastidial ppGpp is suggested to function both as a local repressor of plastid gene expression and as a global regulator of nuclear transcriptional networks under N deficiency. This dual role enables plants to optimize metabolic allocation, suppress costly processes, and activate adaptive developmental and stress responses. Future work should dissect the molecular intermediates of ppGpp signaling and its integration with other pathways.

## Data Availability

The datasets presented in this study can be found in online repositories. The names of the repository/repositories and accession number(s) can be found in the article/**Supplementary Material**.
