# Supplementary material for: Loss of the plastidial signaling molecule, ppGpp, accumulation alters nuclear gene expression during nitrogen starvation
Source: Front Plant Sci. 2026 Apr 20;17:1775338. doi: 10.3389/fpls.2026.1775338 (PMC13136153; doi:10.3389/fpls.2026.1775338)
Supplement: Supplementary file 1 [file Presentation1.pdf]

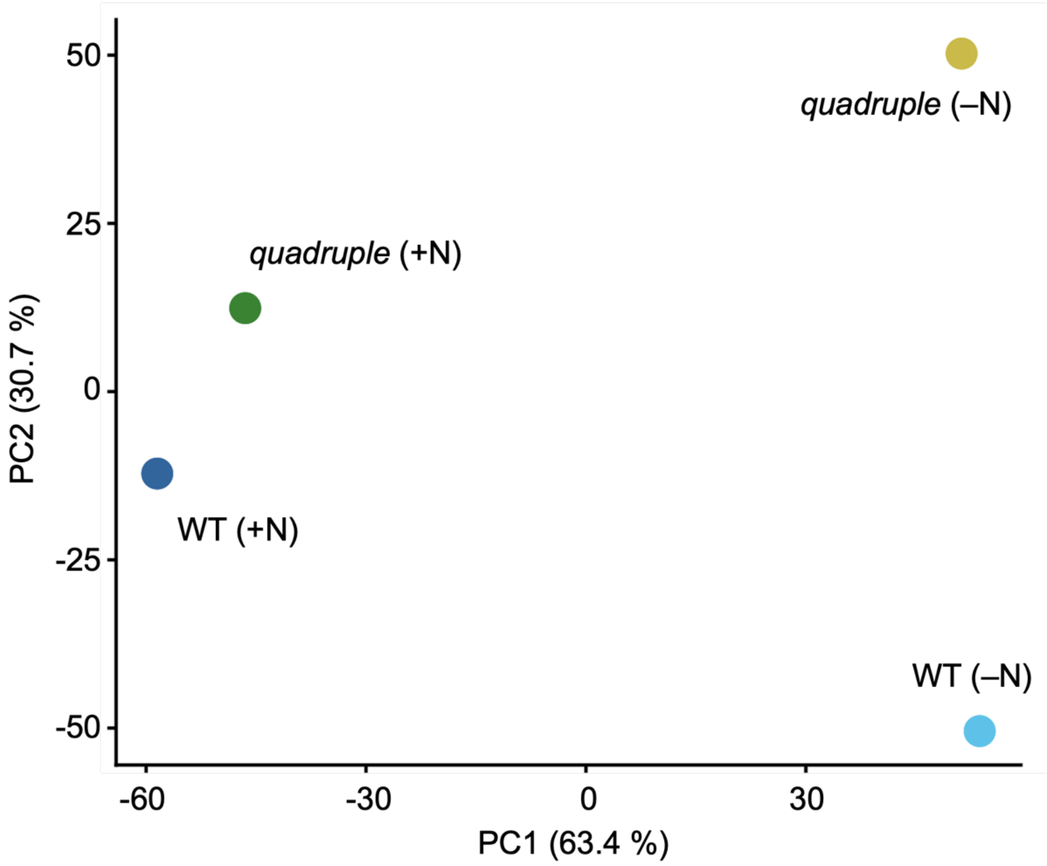

Figure S1: PCA of RNA-seq data.

PCA was performed using log_2-transformed transcript per million (TPM) values. The analysis was conducted on the top 5,000 most variable genes across samples. PC1 and PC2 explained 63.4% and 30.7% of the total variance, respectively.

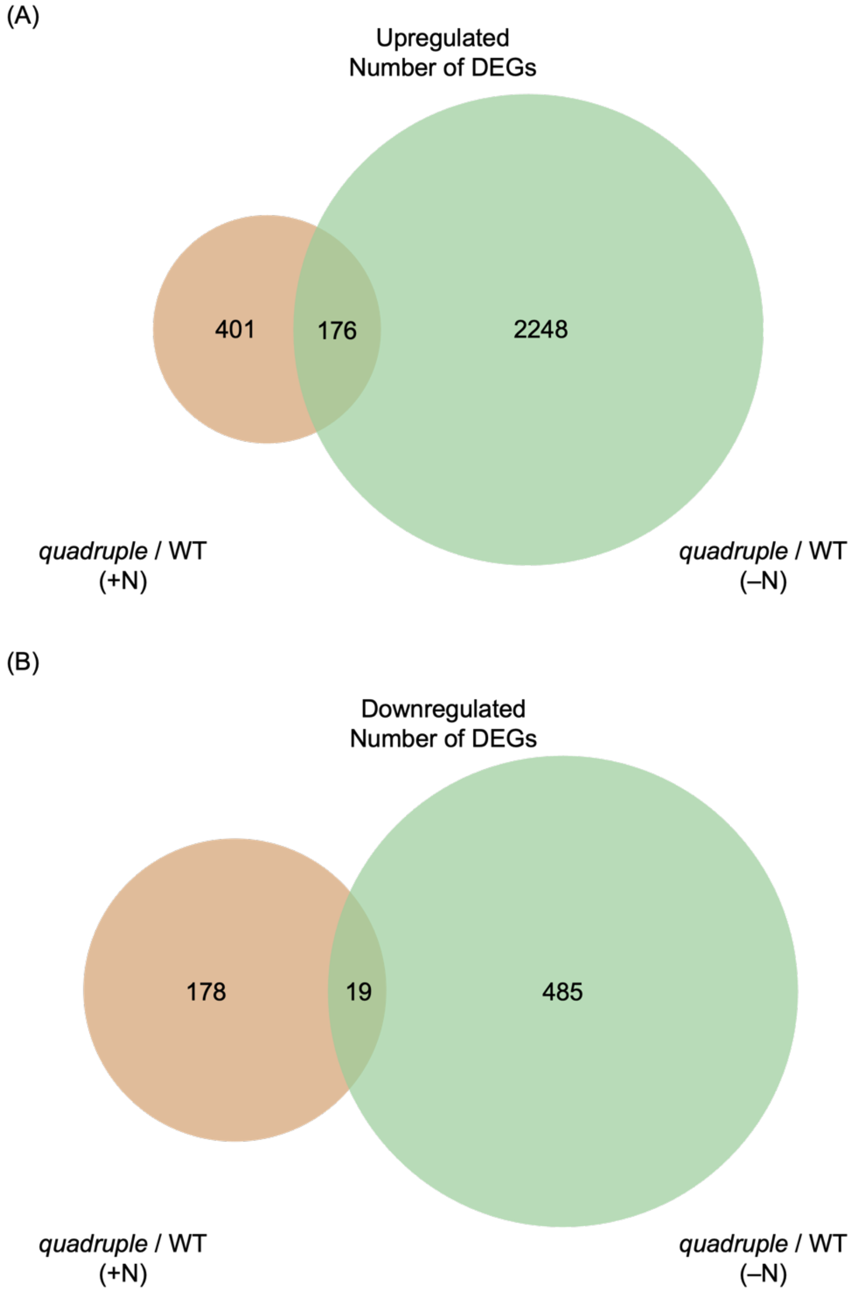

Figure S2: Venn diagrams of upregulated and downregulated genes identified in Fig. 2A and B.

Venn diagrams showing the overlap of DEGs between the comparisons of *quadruple* / WT under (A) nitrogen-sufficient (+N) and (B) nitrogen-deficient (−N) conditions. The overlap between gene sets was statistically significant (hypergeometric test, upregulated genes: $P = 9.35 \times 10^{-64}$; downregulated genes: $P = 1.78 \times 10^{-10}$).

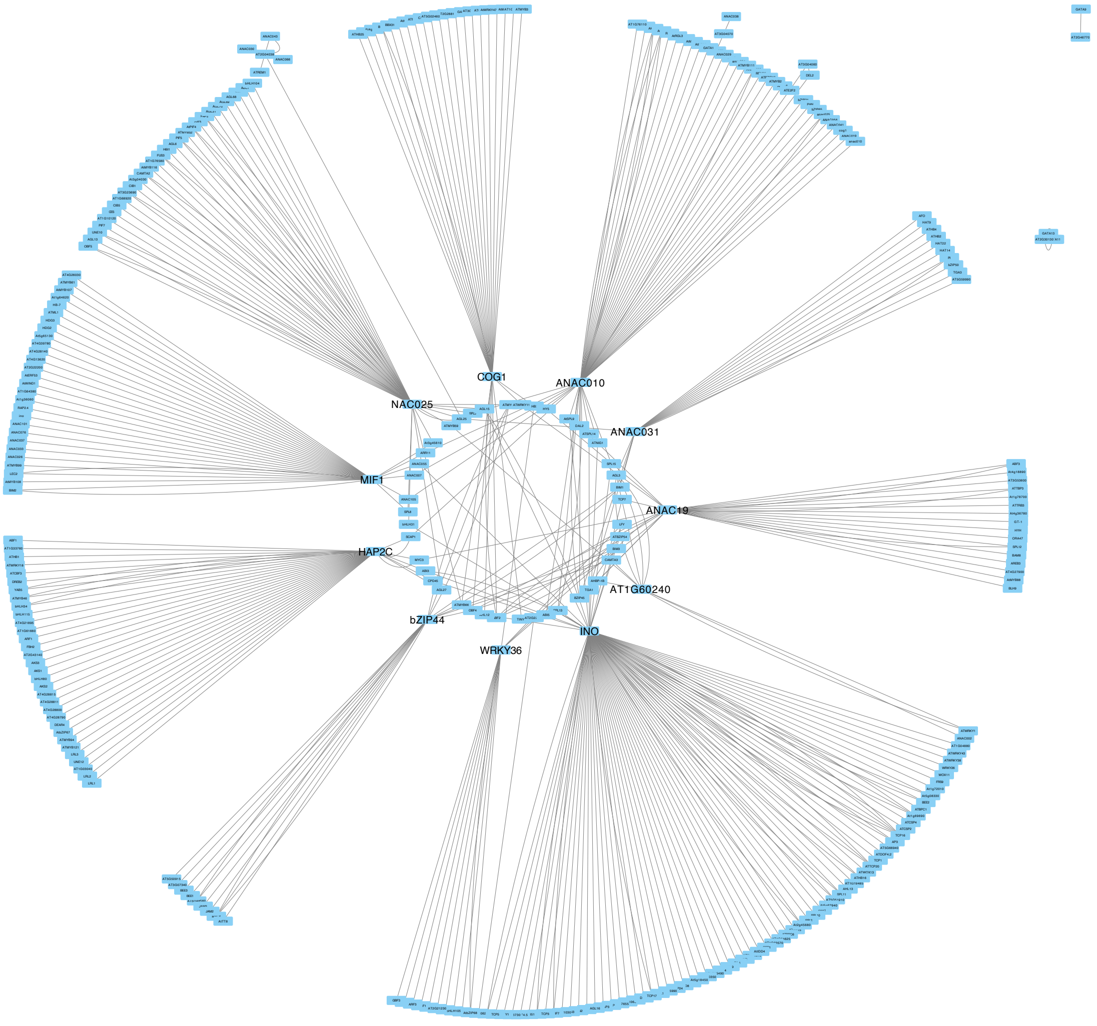

Figure S3:The complete transcriptional network of genes uniquely upregulated in WT under nitrogen-deficient conditions.
